# Radiographic Measurements Correlate to Isolated Posterolateral Corner (PLC) Injury in a Novel Cadaveric Model

**DOI:** 10.7759/cureus.43287

**Published:** 2023-08-10

**Authors:** Joseph D Henningsen, Scott Huff, Andrew Reichard, Andrew Froehle, Anil Krishnamurthy

**Affiliations:** 1 Orthopaedic Surgery, Wright State University, Dayton, USA; 2 Kinesiology and Health, Wright State University, Dayton, USA; 3 Orthopaedic Surgery, Veterans Affairs Medical Center, Dayton, USA

**Keywords:** knee anatomy, knee injuries, diagnostic testing, cadaveric study, postero-lateral knee dislocation

## Abstract

Introduction: Injury to the posterolateral corner (PLC) of the knee often requires surgical reconstruction. There remains no consensus on treatment for PLC injury, and, therefore, it is imperative to have a reproducible injury model to improve the general knowledge of PLC injuries. A novel cadaveric model of isolated PLC injury is proposed and evaluated using radiographic parameters as well as gross dissection.

Material and methods: All protocols were reviewed by the Human Investigation and Research Committee of the home institution and were approved. Translational force in a defined posterior and lateral direction was applied to cadaveric native knees to induce PLC injury. Varus and recurvatum stress fluoroscopic imaging was obtained of each specimen before and after the injury model. Lateral joint distance and recurvatum angle after stress was measured on each image via picture archiving and communication software (PACS) imaging software. After the injury model, injured structures were assessed via saline loading and gross dissection. Any specimens found to be fractured were excluded from the analysis of stress radiography.

Results: A total of 12 knees underwent testing and 6/12 successfully induced PLC injury without fracture. The lateral capsule was torn in every specimen. The popliteofibular ligament (PFL) was torn in 83% of specimens and the fibular collateral ligament (FCL) in 66.7% of specimens. The median lateral gapping after injury under varus stress radiography was 5.39 mm and the median recurvatum angle after injury was 14.25°. Radiographic parameters had a direct relationship with a number of structures injured.

Conclusions: This is the first successful cadaver model of PLC injury. The lateral capsule was injured in every specimen emphasizing the importance of this structure to the PLC.

## Introduction

Injuries to the posterolateral corner (PLC) of the knee can lead to significant rotational, varus, and hyperextension instability requiring extensive surgical reconstruction [1-5]. The PLC structures are made up of both static and dynamic stabilizers and PLC instability has been found to have a wide array of injuries to the individual anatomic components [6]. The rare nature and anatomically heterogeneous nature of PLC injury make clinical studies on the topic difficult. Further complicating the matter, diagnosis of PLC instability can be difficult in the clinical setting as it often occurs in the setting of concomitant cruciate ligament injury. Ancillary tests such as stress radiography and MRI are commonly utilized but have been shown to have their own limitations [7-10].

Previous cadaveric studies have provided data to aid in the clinical diagnosis of PLC injury by examining how the major identifiable PLC structures contribute to lateral “gapping” of the joint line under stress radiography [1, 11-12]. These studies have shown that the main static stabilizers of the knee, including the fibular collateral ligament (FCL), popliteofibular ligament (PFL), and popliteus tendon (PLT), can be assumed to be incompetent with 4.0 mm of gapping seen on stress radiography [12]. However, these studies failed to incorporate capsular injury into their methodology which is the most commonly injured structure seen in PLC instability. Also, these studies involved surgically sectioning and incising components of the PLC which is reproducible but poorly replicates clinical PLC injury which is a result of external forces loaded to the native knee.

 A model of varus-hyperextension injury that can successfully induce isolated PLC injury would allow for a correlation of stress radiography to injury severity that accounts for PLC injury heterogeneity. Hyperextension cadaveric models have been used to study cruciate ligament injuries [13] and patellofemoral ligament injuries [14] in the past and we propose a similar cadaveric model to study isolated PLC injury. To the author's knowledge, this is the first cadaveric model inducing PLC injury via a force-loading mechanism. Using this model, we evaluated previously published varus stress radiographic parameters to aid the clinician in the diagnosis of all grades of PLC injury. Specifically, our goal is to describe the relationship between stress radiography and anatomic structures injured after our PLC injury model. We then used the results of the model to propose the most likely progression of PLC injury within our model. It was hypothesized that the deeper lateral structures would be injured first in the form of capsular injury and progress externally from there ending with the FCL. This article was previously presented as a poster at the 2021 Mid-America Orthopaedic Association Meeting on October 2, 2021.

## Materials and methods

Prior consent by all donors was obtained upon receipt of cadaver donation to Premier Health Miami Valley Hospital for educational and research purposes. All protocols were reviewed and approved by the Premier Health Miami Valley Hospital Human Investigation and Research Committee (# 19-094). Twelve total fresh frozen cadaveric lower extremities from seven cadaver specimens were used in this study. Inclusion criteria required that each knee has no instability on exam and no surgical scars or knee implants seen on fluoroscopy. The limbs studied were from cadavers donated to the home institution for educational and research purposes, with prior consent by all donors. Knees were excluded from analysis if no injury was found on gross dissection or if tibial plateau fracture was found on imaging or gross dissection.

Cadaveric varus-hyperextension model

**Figure 1:**
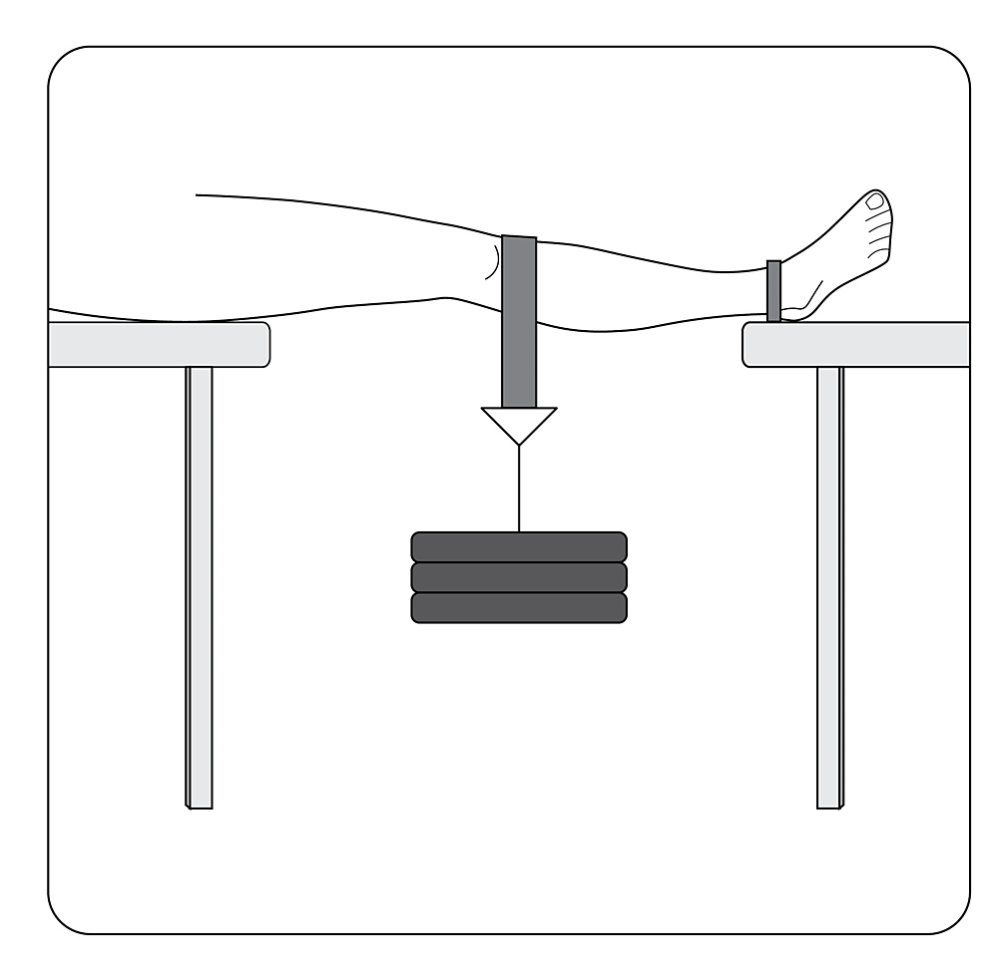
Posterolateral force loading mechanism used to produce high energy/fast strain rate knee posterolateral corner injury.

To induce a PLC injury, the ankle and hip of each limb were secured in separate brackets that allow for distraction through the knee and bending in the plane of gravity. All other translation and rotation were prevented. Each limb was arranged in an orientation so that a horizontal plane was created between the medial border of the patella and the medial epicondyle of the femur. This orientation allowed for the gravitational force applied to the distal femur to induce a varus-hyperextension moment through the knee leading to maximal stress being placed on the PLC. The varus-hyperextension stress was applied through a harness suspending a weight across the distal femur at the level of the superior pole of the patella (see Figure 1).

The weight was supported by a platform that dropped away so that the force on the PLC was applied at the acceleration of gravity when the supporting platform was removed. Weight was increased incrementally until a PLC injury of the limb was suspected, based on visual deformity and physical exam by two researchers. Weight required to induce PLC injury of the limb ranged from 76 kg to 102 kg corresponding to a range of 750-1001 N of force. 

Stress fluoroscopy

**Figure 2:**
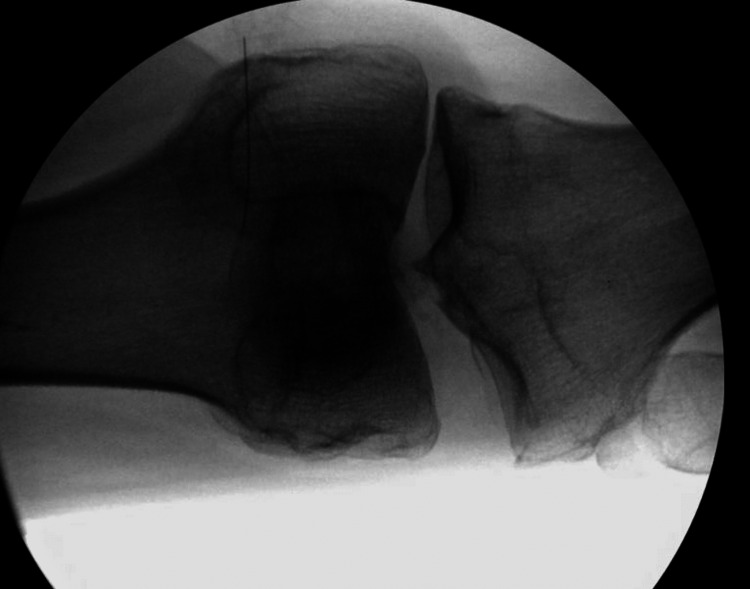
Example fluoroscopic image of varus stress testing where lateral “gapping” was measured both before and after posterolateral corner injury was induced.

Fluoroscopic imaging was acquired using a fluoroscopy C-arm (MiniView 6800 mobile imaging system; GE Healthcare, Milwaukee, WI) to obtain measurements both pre and post PLC injury model. Fluoroscopic views included anterior posterior (AP) and lateral views in full extension, 30 degrees of flexion, and 90 degrees of flexion. Stress fluoroscopy included a manual varus stress test at 30 degrees of flexion and a recurvatum test as described by LaPrade and Terry [3, 11]. A radio-opaque marker was placed in the imaging field just above the joint line to serve as a magnification correction guide. All images were uploaded onto picture archiving and communication software (PACS) for analysis and measurement. Varus stress radiography assessed with PACS software allowed for measurement of lateral joint space both pre- and post-model. Measurement of the perpendicular distance between the most distal lateral femoral condyle and the lateral tibial plateaus as described by LaPrade and Terry previously (see Figure 2) [12].

**Figure 3:**
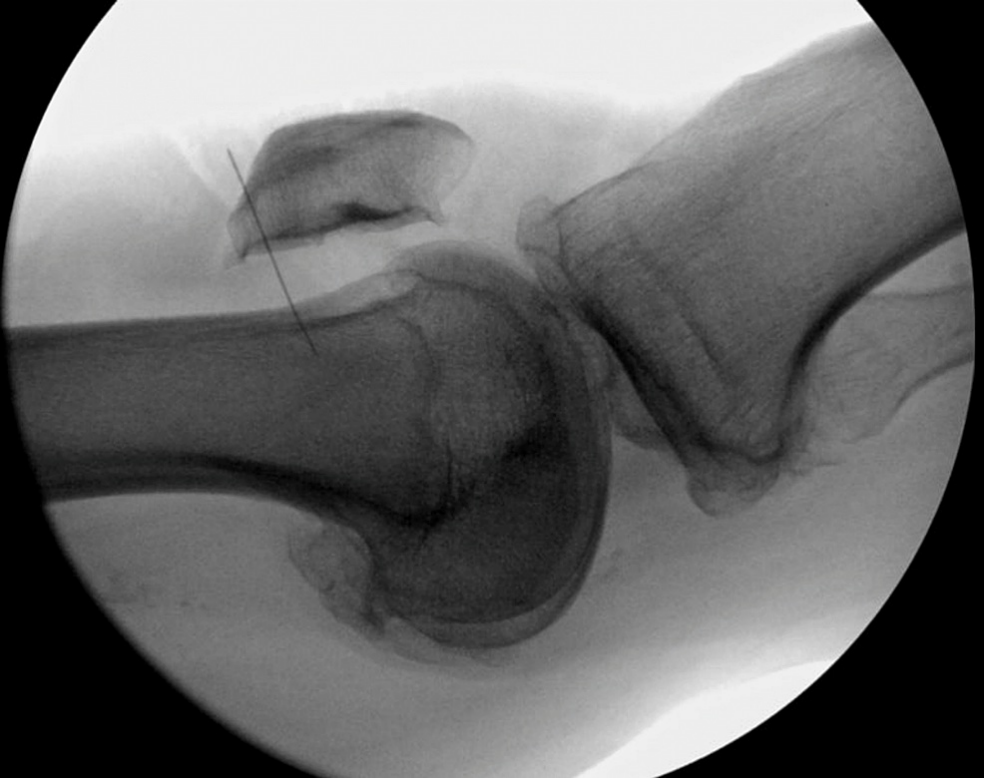
Example of fluoroscopic image of hyperextension stress testing where recurvatum angle was measured both before and after posterolateral corner injury was induced.

The difference between these measurements pre- and post-injury model was assigned as the lateral joint space “gapping.” Hyperextension angle was measured on each cadaver pre- and post-injury model as well. Assessment of the lateral radiograph during the recurvatum test measured the recurvatum angle (RA) between the femoral medullary canal and the tibial medullary canal (see Figure 3).

 Specimen dissection

Following the collection of radiographic images, each specimen underwent capsule integrity testing followed by dissection to assess what structures were injured in the PLC and elsewhere. Capsule integrity testing involved loading joint capsules with saline via percutaneous injection to assess for capsular injury prior to any incisions. The knee was then dissected using a fascial splitting posterolateral approach. The capsular injury was determined to be present if the fluid was seen to drain from the knee prior to any capsulotomy. The PLC structures, including the static stabilizers of the knee (FCL, PLT, and PFL), were assessed for the presence and level of injury. Assessment of injury to the biceps femoris tendon (BFT), iliotibial (IT) band, lateral gastrocnemius origin, and the common peroneal nerve (CPN) were documented as well.

Data analysis

Datasets are presented with descriptive statistics including median and range for continuous measurements between specimens. Ordinal variables, such as whether or not a structure is injured, are presented as frequencies of specimens with the corresponding injury. Measurements were compared pre- and post-injury using Wilcoxon-Mann-Whitney tests. Statistical significance was set to α=0.05.

## Results

Twelve unique cadaveric specimens were put through our varus-hyperextension injury model. Two specimens resulted in no structures injured on dissection post-model. Four specimens were excluded due to tibial plateau fractures to avoid confounding of stress radiography data from fracture motion.

 Results of stress testing pre-injury model are presented in Table 1 including lateral joint space under varus stress and recurvatum angle under hyperextension stress. Table 2 shows which structures were found to be torn in each specimen with corresponding differences in stress radiography measurements post-injury compared to pre-injury stress radiography reported as lateral “gapping” and change in recurvatum angle. Six total specimens could be included in post-injury stress measurement. Post-injury measurements were found to be statistically significantly different for both lateral joint space under varus stress [Pre: 7.9 mm {5.8 mm-12 mm}, Post: 14 mm {9.0 mm-18 mm}, p < 0.05] and recurvatum angle under hyperextension stress [Pre: 3.7° {-8.3°-8.3°}, Post: 16.3° {6.3°-47°}, p < 0.05] compared to pre-injury measurements. The median lateral gapping seen post-injury was 5.4 mm with a range of 1.6-11 mm. The change in recurvatum angle had an even wider range from -2.0° to 44.2° with a median of 14.3°. Frequency of tears found in each individual anatomic structures is summarized in Table 3. The lateral capsule was torn in 75% specimens and the PFL was the next most commonly torn structure at 63% followed by the FCL at 50%.

**Table 1:** Pre-injury radiographic measurements for each cadaver specimen.

Limb ID number	Pre-injury lateral joint distance (mm)	Pre-injury recurvatum angle (° , positive in flexion)
001	12	7.0
002	9.3	8.3
003	6.8	5.9
004	5.8	-8.3
005	7.2	7.0
006	7.1	2.6
007	7.1	4.8
008	8.9	1.5
009	7.4	-2.9
010	8.9	2.4
011	8.3	0.90
012	9.1	4.8
Median	7.9	3.7
Range	6.2	16.6
Standard deviation	1.6	4.7

**Table 2:** Summary of cadaver model findings including torn structures and radiographic measurements post-injury. PCL, posterior collateral ligament; PLT, popliteus tendon; PFL, popliteofibular ligament; FCL, fibular collateral ligament; LGT, lateral gastrocnemius tendon; LBFT, long biceps femoris tendon *Knees without injury did not undergo post-model stress radiography analysis; **Knees were found to have tibial plateau fracture and were excluded from analysis

Limb ID	Torn structures post-model	Lateral "gapping" (mm)	Δ Recurvatum angle (°, positive in flexion)
001	No injury	NA*	NA*
002	Lateral capsule, PFL	4.9	-2.0
003	Lateral capsule, PFL, FCL, LGT	8.1	20.
004	Tibial plateau fracture	NA**	NA**
005	Lateral capsule	5.9	9.1
006	Lateral capsule, PFL, FCL, LGT, BFT	11	44.
007	No injury	NA*	NA*
008	Tibial plateau fracture	NA**	NA**
009	Lateral capsule, PFL, FCL	1.6	15
010	Lateral capsule, PFL, FCL,	3.7	14
011	Tibial plateau fracture	NA**	NA**
012	Tibial plateau fracture	NA**	NA**
Median		5.4	14
Range		9.4	46
Standard deviation		3.3	15

**Table 3:** Frequency of injured structrues post-injury model. FCL, fibular collateral ligament; PFL, popliteofibular ligament

Structure	Number of tears	Percentage of specimens (%)
Lateral capsule	6	75
PFL	5	63
FCL	4	50
Lateral gastrocnemius tendon	2	25
Biceps femoris tendon	1	13
Popliteus tendon	0	0
Common peroneal nerve	0	0

## Discussion

Posterolateral corner injury to the knee remains a difficult topic to study and diagnose as illustrated by the heterogeneity of our anatomic structures injured in our highly controlled laboratory setting. A reproducible cadaver model such as the one proposed in this article can allow for the study of how and why these injuries are so heterogeneous and difficult to treat. A cadaveric varus-hyperextension model is described and successfully induced isolated PLC injury in six out of twelve specimens. Of note, capsular tears were seen in 75% of specimens (100% of injured specimens) which agrees with clinical study results shown by LaPrade and Terry where 75% of patients in that study had tears of the capsulo-osseous layer of the iliotibial band and 58% had other capsular injuries [3]. These results further highlight the importance of capsule integrity to posterolateral knee stability. In one of our specimens, the lateral capsular injury alone had a direct result of lateral gapping measuring 5.9 mm and significant instability on examination. This is in contrast to stress radiography described in previous literature which stated that lateral gapping greater than 4.0 mm is predictive of complete disruption of the FCL and PFL [12]. Comparison of our model to these previous studies may be limited because previous studies used an in vitro PLC injury induced by surgically sectioning the components of the PLC while our model uses an external varus-hyperextension force to more closely model clinical PLC injury. Furthermore, these studies did not evaluate capsular contribution to stability which alone could explain this discrepancy. Our results illustrate that biomechanical testing of the capsular contribution to posterolateral stability is warranted particularly when considering the diagnostic utility of lateral gapping on stress radiography.

 Extrapolation of our varus-hyperextension model data allows for the creation of hypotheses applicable to multi-ligamentous knee injuries. Our model isolates posterolateral forces applied to the knee in extension and our results highlight the importance of the lateral capsule in knee stability in this position. While the posterior cruciate ligament is a key stabilizer to posterolateral forces in flexion; our model supports that the lateral capsule contribution is important in extension which leads to the hypothesis that the lateral capsule may be a secondary stabilizer in flexion. The authors hypothesize that the lateral capsule is the first structure to fail in PLC injury which could lead to the progression of more superficial structures including the FCL and PCL. Further studies are needed to test and confirm this hypothesis. It is important to note that our model is an isolated PLC injury model since no rotational force was applied to the cadaver specimen as was illustrated given no cadaver specimen was found to have cruciate ligament injury. Only 33% of patients who present with PLC injury have been shown to have an isolated injury on MRI [3]. Our results are, therefore, only directly applicable to a subset of clinical patients, however, focusing on a single force vector allows for the creation and evaluation of a reproducible injury model. Further study and innovation would be needed to create a reproducible model that assesses concomitant rotation injuries with PLC injuries.

 Our methods detail a novel model of inducing varus-hyperextension force upon a cadaver knee in order to induce PLC injury. It is of note that our model was limited by the fact that only six out of 12 specimens had a successfully induced PLC injury in the absence of fracture. The high rate of tibial plateau fracture from the model could be explained by anatomic variation between specimens, but this also warrants further study. Based on our results, certain specimens were more susceptible to tibial plateau fracture. Specimens that suffered tibial plateau fracture had less preoperative extension than those with intact tibial plateaus (-0.3° vs 4.9°). It is hypothesized that flexion contractures in specimens before the injury model contributed to a specimen being more susceptible to tibial plateau fracture from the injury model. Therefore, it is recommended that any reproduction of these methods uses cadavers without flexion contractures to maximize the efficiency and reproducibility of the model. We also noticed significant variability in the Δ Recurvatum Angle measurements post-model as illustrated by the large range and standard deviation values (range: 46°; standard deviation: 15°). This high degree of variability of our sagittal plane measurements provides motivation to further optimize the injury model to answer more clinical questions. The authors hypothesize that a more standardized ability to control the amount of flexion/extension of each specimen within the injury model could reduce the dispersion of the Δ Recurvatum Angle measurements and make the results more reproducible. Also, control over the flexion/extension positioning would allow for a more detailed study of the relationship between leg positioning on PLC injury using the model. 

The results of this study should be interpreted within the framework that cadaver biomechanical studies incompletely model viable tissue. Specifically, our model involves rapid loading of a static extremity. In a clinical injury to the PLC, dynamic muscular contraction alters the forces and stability that the PLC experiences. In our cadaver model, all muscular stabilizers of the PLC are static, and interpretation of muscular injury to the popliteus, biceps femoris, and lateral gastrocnemius has limited applications to clinical injury. Furthermore, stress examination in the acute setting is often limited due to pain and may require examination under anesthesia in order to produce accurate and repeatable results in the clinical setting. Finally, out of necessity for reproducibility, we limited our model to translational force through the knee joint only. Clinical PLC injury occurs via three-dimensional loading under dynamic conditions including complex rotational kinematics. This model should always be interpreted through the lens that it is a reproducible simplification of a complex, dynamic system. 

## Conclusions

A novel, reproducible cadaver varus-hyperextension model is described to study PLC injury. Some 100% of included specimens with PCL instability had an injury to the lateral capsule. Stress radiography of these specimens showed that significant lateral gapping on varus stress radiography can be seen with isolated lateral capsule injury indicating that the lateral capsule may play a more significant role in PLC stability than what has been previously described. The model described can be used as a template for future research into PLC injury including the study of the effects of sagittal alignment on PLC injury, the sequence of PLC injury, and more accurate diagnosis of PLC injury. 
